# Ergonomics of 3D‐Exoscope Versus the Operating Microscope in Otologic Surgery

**DOI:** 10.1111/ans.70278

**Published:** 2025-08-04

**Authors:** Ankit Ajmera, Raewyn G. Campbell, Jennifer W. Y. Lee, Samuel J. McGinness, Payal Mukherjee

**Affiliations:** ^1^ ENT Department Sydney Adventist Hospital Sydney Australia; ^2^ Faculty of Medicine, Health and Human Sciences Macquarie University Sydney Australia; ^3^ Faculty of Medicine and Health University of Sydney Sydney Australia; ^4^ Institute of Academic Surgery Royal Prince Alfred Hospital Sydney Australia

**Keywords:** 3D‐exoscope, ergonomics, microscope, musculoskeletal pain, otology

## Abstract

**Introduction:**

Surgeons experience one of the highest rates of work‐related musculoskeletal disorders, with otologists identified as a high‐risk specialty. This is commonly attributed to the frequent use of the operating microscope, as it often necessitates prolonged, ergonomically unfavourable, awkward postures. Robotic 3D‐exoscopes have emerged as a potential tool in improving surgical ergonomics without compromising on visualisation. This study compares the ergonomic risk associated with both devices.

**Methods:**

A prospective, single centre, ergonomic analysis was conducted on otolaryngologists performing major otologic surgery using the OPMI Sensera operating microscope (Zeiss) and RoboticScope 3D‐exoscope (BHS Technologies). Ergonomic risk was evaluated using the Rapid Upper Limb Assessment (RULA) tool and a questionnaire based on the NASA Task Load Index.

**Results:**

Eighteen cases were performed; nine using the 3D‐exoscope and nine using the operating microscope. Use of the 3D exoscope significantly reduced ergonomic risk, with a lower mean RULA score (4.0) compared to the microscope (5.9). Subjectively, surgeons reported reduced physical strain, effort and weakness when using the 3D‐exoscope compared with the microscope, with similar cognitive and mental workload. Image quality, magnification, colour contrast, ease of use and usability of the 3D‐exoscope were rated positively. Further, the 3D‐exoscope was also rated highly for its educational value.

**Conclusion:**

3D‐exoscopes appear to be more ergonomically favourable and suitable when compared to the operating microscope for major otologic surgery. Continued development and larger scale studies are warranted to improve ergonomics in otologic surgery and can also inform ergonomic improvements across other surgical specialties.

## Graphical abstract

Microscope use in otologic surgery is associated with a high degree of ergonomic risk and musculoskeletal pain. A prospective comparative study was undertaken demonstrating a 3D‐exoscope has improved ergonomics compared to the operating microscope. Ongoing development is required to improve the ergonomics in otologic surgery on a larger scale.

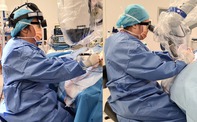

## 1 Introduction

Ergonomics focuses on reducing the risk of developing injury while improving efficiency, and is important for surgeons to consider [1]. Literature has demonstrated that healthcare workers, and in particular surgeons, have among the highest rates of work‐related musculoskeletal disorders (WRMDs) in the world, and that surgical ergonomics lags behind most other occupations [2, 3]. WRMDs are especially common in otolaryngology, with 76%–97% of otolaryngologists reporting these symptoms [4, 5]. This rate is high compared to robotic surgeons, who have one of the lowest rates within the surgical subspecialty at 56% [6], and the general Australian population at 15% [7]. Furthermore, WRMDs also affect trainees, with up to 93% of otolaryngology trainees with less than 5 years of experience reporting these symptoms [8].

The most common sites of musculoskeletal pain for otolaryngologists involve the neck, back and shoulders [4, 9, 10]. In particular, as neck flexion increases, the strain on the cervical flexor muscles increases, with up to 27 kg of additional load placed on the cervical spine at 60° of neck flexion [2, 11]. Clinicians with musculoskeletal pain report lower job satisfaction, higher rates of burnout, and are more likely to take early retirement and injury‐related leave, leading to greater societal costs [3, 12, 13]. This is concerning given the current medical workforce shortage in Australia, with approximately 1.9 otolaryngologists per 100 000 population, of whom 25.7% are over 60 years of age [14]. Therefore, prompt intervention is needed.

Otologists, in particular, face several challenges in achieving an optimal surgical view during microscopic surgery. When using a microscope, otolaryngologists spend up to 73% of their time in an ergonomically high‐risk neck position and are more static with greater neck and back flexion when compared to using a head up display [15, 16, 17]. Microscopic otologic surgery carries ergonomic risk as it demands repetitive, fine motor movements within a confined environment while maintaining static and awkward postures for prolonged periods [1, 15, 18]. Surgery also carries a high cognitive load, further increasing the ergonomic risk [19].

**FIGURE 1:**
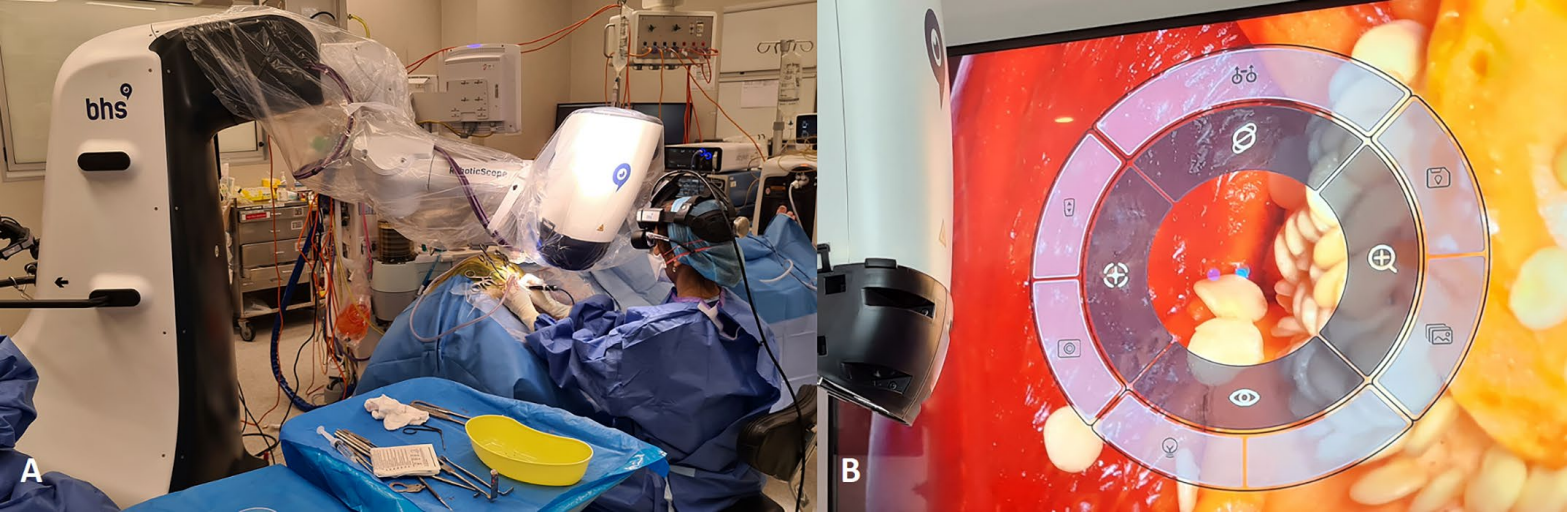
(A) Theatre layout with the RoboticScope in action during a mastoidectomy; (B) Display control panel during activation of the foot pedal when viewing a capsicum during a practice session.

There is an evolution of early literature comparing the ergonomic risk of 3D‐exoscopes to traditional microscopes in otolaryngology. A 3D‐exoscope is a digital robotic microscope positioned over the surgical field, with the images displayed either on a monitor or on a head mounted device (HMD). This allows the surgeon greater freedom of movement without disrupting the surgical view, allowing for rest and reduced time spent in static or non‐ergonomic postures. The RoboticScope (BHS Technologies Ltd., Innsbruck, Austria) utilises a HMD to provide a 3D image directly to the wearer. The field of view is adjusted using a foot pedal and head movements. (Figure 1) An additional HMD can be worn by an assistant and additional monitors can be connected.

This study undertook an ergonomic assessment comparing the RoboticScope to a traditional microscope during major otologic procedures.

## 2 Methods

A prospective pilot study was conducted to analyse the ergonomic position of otologists performing major otologic surgery in a single centre. Nine procedures were undertaken with an OPMI Sensera microscope (Carl Zeiss Pty Ltd., Oberkochen, Germany) and nine with the 3D‐exoscope to assess the feasibility of the 3D‐exoscope in improving ergonomics (Table 1). Cases that were already booked for theatre at the time of the study were included. Photographs were taken at 30‐min intervals when either the 3D‐exoscope or the microscope was used. Notably, the microscope lacked an adjustable eyepiece and all procedures were performed using the Surgistool (Stryker Pty Ltd., Michigan, USA) adjustable chair without arm supports.

**TABLE 1:** Case mix.

Procedure	Operating microscope	3D‐exoscope
Cochlear implant	3	5
Removal of exostosis	2	1
Modified radical mastoidectomy for Cholesteatoma	1	1
Canal wall up mastoidectomy for cholesteatoma	2	2
Stapedectomy for otosclerosis	1	0

The photographs were analysed using the Rapid Upper Limb Assessment (RULA) score to measure ergonomic risk [20]. The RULA tool is an objective and validated instrument used in multiple studies, and provides an overall ergonomic risk score out of 7 with an associated recommended action [1, 4]: scores of 1–2 are deemed negligible ergonomic risk with no action required; 3–4 are low ergonomic risk with further investigations needed; 5–6 are deemed moderate ergonomic risk with action needed soon; and a score of 7 demonstrates a high ergonomic risk with investigations and immediate changes recommended. Average RULA scores were compared between the two modalities.

A questionnaire based on the National Aeronautics and Space Administration (NASA) Task Load Index [21] with a 9‐point Likert scale was completed by the surgeons at the end of each procedure. The NASA Task Load Index is a validated tool that provides a subjective analysis of workload, effort, performance, frustration and mental, physical and temporal demands [22]. This was expanded to include fatigue, physical strain and weakness and an assessment of the 3D‐exoscope itself, with a focus on ease of use, image quality, and educational opportunities. Responses were scored across a 9‐point scale (i.e., ‘strongly disagree’ scored 1 point, ‘neutral’ scored 5 points and ‘strongly agree’ scored 9 points). Scores were compared with a two‐tailed Mann–Whitney U‐test using Statistical Product and Service Solutions software version 29 (SPSS; IBM, Illinois, USA) with significance set at *p* < 0.05.

## 3 Results

Three otologists at a single centre were enrolled in the study after spending time training with a company representative. (Table 2).

**TABLE 2:** Surgeon demographics.

*N*	3
% male	0.33%
% right‐handed	100%
Age (mean; range)	40.67 (38–45)
Years practicing as a consultant (years mean; range)	7.67 (5–11)
Height in metres (mean; range)	1.68 (1.61–1.81)
BMI (kg/m^2^)	24.61 (18.13–28.22)

### 3.1 RULA Score

A reduction of ergonomic risk was observed with the use of a 3D‐exoscope compared to the operating microscope. The mean RULA score for the microscope was 5.9 out of 7 (range 4–7), indicating a moderate ergonomic risk. The RULA guidelines thus recommend that changes be implemented soon to reduce the risk of developing WRMDs. In comparison, the mean RULA score for the 3D‐exoscope was 4 out of 7 (range 3–5), indicating a low ergonomic risk.

### 3.2 Questionnaire

#### 3.2.1 Mental and Physical Demand

**FIGURE 2:**
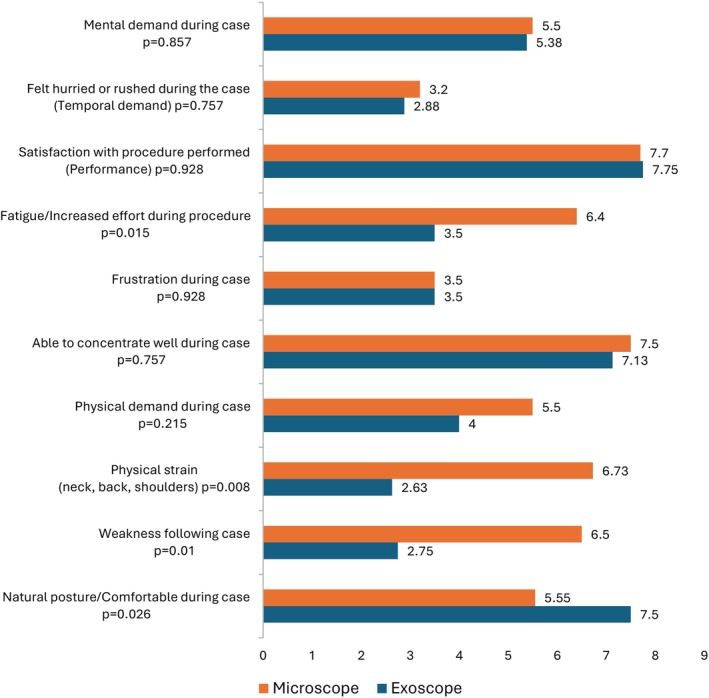
Graph of mean Likert scores comparisons.

There was a statistically significant reduction in the scores between the 3D‐exoscope and the operating microscope for fatigue and effort (mean score 6.4 and 3.5 respectively, *p* = 0.015), physical strain (mean score 6.73 and 2.63 respectively, *p* = 0.008) and weakness (mean score 6.5 and 2.75 respectively, *p* = 0.01). Surgeons also reported a more natural posture with the 3D‐exoscope compared to the microscope (mean score 5.55 compared to 7.5 respectively, *p* = 0.026). There were no statistical differences with mental demand, ability to concentrate or satisfaction with the operation, indicating no additional cognitive burden is associated with using the robotic device (Figure 2).

#### 3.2.2 Functionality

**FIGURE 3:**
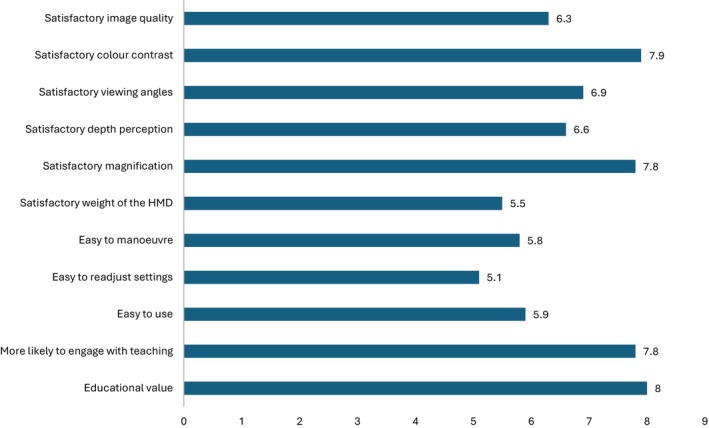
Graph of mean Likert scores on RoboticScope usability.

Based on the Likert scores, surgeons reported good image quality (mean 6.3), magnification (mean 7.8), viewing angles (mean 6.9) and colour contrast (mean 7.9) when using the RoboticScope. It took time to become familiar with adjusting and manoeuvring the 3D‐exoscope, and the depth perception was noted to be exaggerated through the HMD, with anatomical structures appearing deeper than in reality. These limitations improved with repeated use. Additionally, surgeons rated highly the educational value of the RoboticScope (mean 8) and felt that it would improve their ability to teach trainees (mean 7.8). Surgeons attributed this to the 3D‐exoscope's ability to provide shared binocular vision and depth perception to both the surgeon and the trainee, an advantage not afforded by the microscope (Figure 3).

### 3.3 Patient Safety

There were no adverse events noted across the nine patients where the robotic device was used. There was one conversion from the 3D‐exoscope to the microscope, which occurred towards the end of a modified radical mastoidectomy to complete the middle ear dissection. This was attributed to the surgeon's unfamiliarity with the device, as it was their first time using the 3D‐exoscope and subsequent cases did not lead to a conversion. As the 3D‐exoscope was used for the majority of the case, data collected and analysed was used until the visualisation device changed.

## 4 Discussion

Otologic procedures are currently performed using either an operating microscope or with endoscopic techniques. While the endoscope provides better visual clarity and ergonomics compared to the microscope, barriers to adoption include a lack of stereoscopic vision, static upper limb postures and reliance on single‐handed surgery [16, 23]. With the evolution of robotics, there has been a drive to integrate this technology into surgery, particularly for its enhancements in visualisation and ergonomics, while also maintaining the ability to perform two‐handed surgery [23]. This has led to the development of robotic 3D‐exoscopes, with several systems now on the market.

This pilot study compares the ergonomic risks of the operating microscope and the RoboticScope 3D‐exoscope during otologic surgery, using both qualitative and quantitative outcome measures. The high prevalence of WRMDs among otolaryngologists is concerning, as it affects surgeons throughout their careers. While awareness is gradually increasing, studies show only 24% of surgeons have any knowledge or training in ergonomics [8]. While ergonomics in surgery still lags behind most other occupations [2, 3], early studies have started to explore strategies to mitigate this risk. These include modifications to theatre set‐up, surgical equipment and the implementation of micro‐breaks [1, 2, 13], however, literature evaluating the impact of the 3D‐exoscope when compared with other modalities is limited.

### 4.1 Ergonomics

**FIGURE 4:**
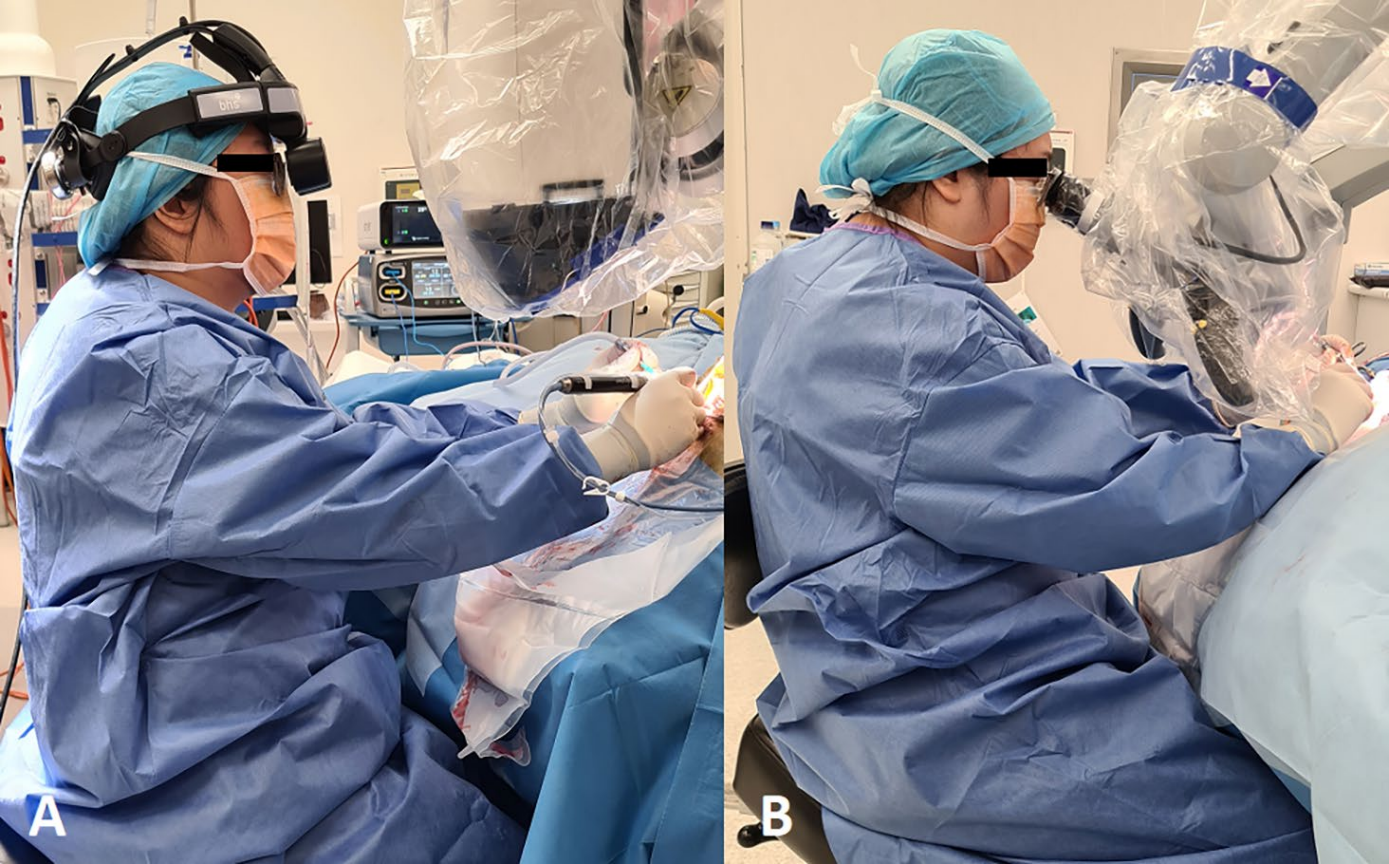
Posture during otologic surgery. (A) RoboticScope, BHS Technologies. (B) OPMI Sensera Zeiss Microscope.

This study demonstrates that surgeons frequently adopt poor postures when using a microscope, which improved with the 3D‐exoscope. There were marked differences in surgeons' postures between the devices (Figure 4). The mean RULA score using the microscope was 5.9 (moderate risk), with some surgeons scoring 7 (high risk; implement change immediately). This can be attributed to the microscope's fixed optical system, which restricts the surgeon's posture, resulting in WRMDs [23]. In contrast, the 3D‐exoscope was associated with a lower mean RULA score of 4 (low risk), with no surgeon demonstrating a high‐risk score. Subjectively, surgeons also reported reduced effort, physical strain and weakness with the 3D‐exoscope. These improvements are consistent with existing literature [18, 24, 25] and can be attributed to the freedom of movement the device affords.

The RoboticScope's HMD, which weighs approximately 900 g, presents an ergonomic consideration. Unlike display monitors, which restrict the gaze and positioning of both surgeons and assistants to facilitate an unobstructed view, the HMD allows greater freedom of movement and thus promotes more neutral neck posture [23, 26]. However, the HMD increases the load on the cervical spine, which can cause additional strain with prolonged use [4]. Furthermore, the RoboticScope is bulkier than the microscope and was noted to create a narrow working space. While it facilitated a more neutral cervical spine posture, the arms and elbows were observed to be more extended, increasing ergonomic risk in this region. This constraint is consistent with other literature [18].

Lin et al. [18] compared the ergonomic risks during otologic procedures performed by an Orbeye exoscope (Olympus, Tokyo, Japan), a microscope, and an endoscope. They found that the RULA scores were lowest in the 3D‐exoscope arm, with the greatest reduction in musculoskeletal pain, while the microscope presented the greatest ergonomic risk.

Crimi et al. [23] compared the ergonomic risk of otology and lateral skull base surgeons using the Synaptive Modus X Exoscope (Synaptive Medical, Toronto, Canada) with the operating microscope. Surgeons reported greater comfort and reduced post‐operative neck and shoulder pain when using the 3D‐exoscope, and there was a 63.3% reduction in the mean RULA score when using the 3D‐exoscope compared to the microscope.

### 4.2 Functionality

Various 3D‐exoscopic systems have been shown to have excellent image quality, magnification, manoeuvrability, and effective angulation, and the findings of this study are consistent with the literature [23, 24, 27]. However, the RoboticScope's wide stereo base produces a pronounced 3D image and an increased depth of the surgical field. While this is more advantageous in wide field‐of‐view cases (e.g., mastoidectomy), until surgeons become more accustomed to the device, it may not completely replace the microscope in procedures involving more narrow or constrained regions (e.g., transcanal procedures).

Furthermore, unlike the microscope, 3D‐exoscopes enable binocular vision for all individuals wearing a HMD, providing an advantage in surgical education. Supervisors are able to closely monitor their trainees in real time, and trainees are able to visualise the surgical anatomy and techniques with depth perception and clear picture quality [28]. This is not always the case with current traditional modalities. Surgeons in our study reported that 3D‐exoscopes offer educational value and felt it would better enhance their abilities to teach trainees. These findings align with existing literature, which has found that surgical supervisors are more inclined to allow trainees to perform more of the operation, and that trainees consider the 3D‐exoscope a superior educational tool compared to the microscope [25, 27, 28].

### 4.3 Healthcare Costs

Change is not possible without considering the economic costs; however, unless ergonomics is prioritised, high rates of WRMDs will continue, straining the healthcare system further. While costs of the microscope and 3D‐exoscopes are comparable [28], investments in ergonomically sound equipment must be weighed against the cost of lost opportunity in providing for the community and reducing WRMDs in a workforce that is already at high risk of developing injuries. This is especially the case given the current workforce shortages and the investments required in training new surgeons.

Any new technology can impact operative time and consequently the overall costs of surgery. In this study, an initial increase in operative time was observed, largely attributed to the set‐up, positioning, and draping of the RoboticScope. This reduced as the theatre team became more familiar with the workflow. To further improve efficiency, one surgeon adapted their surgical approach by completing all steps not requiring magnification prior to using the RoboticScope, thereby minimising the cumulative impact of repositioning the device. No extra theatre time was allocated for cases performed with either technique in this study. This is supported by evidence in the literature, which reports comparable surgical times with microscopic and 3D‐exoscopic systems [24, 29].

### 4.4 Limitations

Limitations of our study include the small sample size, lack of blinding and randomisation, and the comparison of only one style of operating microscope and 3D‐exoscope. The surgeon's muscle force and strain may be better assessed using surface electromyography, artificial intelligence, or smart technology. A further limitation is the learning curve needed with the 3D‐exoscope, particularly with the depth perception and ocular controls on the HMD; however, this may not be a constraint with other 3D‐exoscopic systems. While training by the company was required prior to the study, only one case required conversion to a microscope. This occurred during the first use of the device by that surgeon, suggesting that the learning curve was not a major limitation to its use. Finally, the participant cohort of early‐to‐mid career surgeons may have created a bias towards reducing the difference found and being more receptive to using newer technologies.

### 4.5 Future Directions

Larger scale trials should be conducted comparing the ergonomic risks in various visualisation modalities (i.e., microscope, 3D‐exoscope and endoscope) over a variety of otologic procedures. Measuring muscle activity and posture with inertial measurement units and videography, along with assessing ergonomics in the outpatient setting, would add to the knowledge and aid in the reduction of WRMD. Further development of robotic devices in surgery to improve their utility will benefit as we continue to integrate technology and device development with patient care.

### 4.6 Workforce Awareness

As a profession, there is an urgent need to increase awareness of ergonomics and resist the culture to ignore discomfort [30]. Several publications, including those from the American College of Surgeons, have recommendations for ergonomic set‐ups [31]. These guidelines include: ensuring an adjustable chair with arm supports, adjusting seat height such that the thighs are parallel to the floor, and a table height that allows the surgeons' elbows to be working at 90°–120° of flexion. Further, appropriate patient and equipment positioning, encouragement of physical activity and microbreaks, increased awareness and policy development and selection of microscopes with articulated eye pieces and a short ocular‐corpus length are important to reduce the risk of WRMDs [1, 2, 32, 33]. Ergonomic practice is also currently lacking in surgical training [2] and studies have demonstrated that incorporating formal training into the curriculum is both feasible and reduces musculoskeletal strain [34, 35, 36].

## 5 Conclusion

This pilot study demonstrates that performing major otologic procedures with a 3D‐exoscope is both feasible and ergonomically advantageous when compared to an operating microscope. WRMDs are common and increasing in surgeons; however, the adoption of improved ergonomic practice in otologic surgery, and across all surgical specialties, has the potential to reduce the risk of injury and optimise patient care.

## Ethics Statement

Ethics was provided by Adventist Health Care Limited. Reference ID: 2023‐010.

## Conflicts of Interest

The authors declare no conflicts of interest.

## Data Availability

The data that support the findings of this study are available from the corresponding author upon reasonable request.
